# Transformation of a Thermostable G-Quadruplex Structure into DNA Duplex Driven by Reverse Gyrase

**DOI:** 10.3390/molecules22112021

**Published:** 2017-11-22

**Authors:** Dawei Li, Qiang Wang, Yun Liu, Kun Liu, Qiang Zhuge, Bei Lv

**Affiliations:** 1 Key Lab of Forest Genetics and Biotechnology, Nanjing Forestry University, 159 Longpan Road, Nanjing 210037, China; qwangntu@gmail.com (Q.W.); sanyecun@163.com (Y.L.); qzhuge@njfu.edu.cn (Q.Z.); 2 Jiangsu Key Laboratory for Biofunctional Molecules, College of Life Science and Chemistry, Jiangsu Second Normal University, Nanjing 210037, China; davylee86@gmail.com

**Keywords:** G-quadruplex, DNA supercoiling, topoisomer, topoisomerase, Atomic Force Microscope examination

## Abstract

Reverse gyrase is a topoisomerase that can introduce positive supercoils to its substrate DNA. It is demonstrated in our studies that a highly thermal stable G-quadruplex structure in a mini-plasmid DNA was transformed into its duplex conformation after a treatment with reverse gyrase. The structural difference of the topoisomers were verified and analyzed by gel electrophoresis, atomic force microscopy examination, and endonuclease digestion assays. All evidence suggested that the overwinding structure of positive supercoil could provide a driven force to disintegrate G-quadruplex and reform duplex. The results of our studies could suggest that hyperthermophiles might use reverse gyrase to manipulate the disintegration of non-B DNA structures and safekeep their genomic information.

## 1. Introduction

Reverse gyrase is a topoisomerase that was found in hyperthermophiles [1,2]. It is believed that reverse gyrase have a special ability to intertwine the double helix of DNA and make it tighter [3,4]. The discovery of reverse gyrase has received considerable attention in the past few years, and various studies have been carried out in order to understand the innate roles of this hyperthermophilic enzyme [4,5,6]. It has been reported that the presence of reverse gyrase is a prerequisite for hyperthermophiles to flourish, while a lack of this enzyme could lead to a retardation in growth that is more striking at higher temperatures [7]. In addition, reverse gyrase is composed of the structural domains of both helicase and type I topoisomerase that enable this hyperthermophilic enzyme to introduce positive supercoiling to its substrate DNA [5,8]. Even though a great deal of information about reverse gyrase and its cellular functions have been obtained, it has not yet been clearly understood up to now why hyperthermophiles utilize this positive-supercoil-introducing enzyme to manipulate and safekeep their genomic DNA.

Non-B DNA structures, on the other hand, represent distortions of the canonical DNA double helix, which could increase the risk of genetic instability and thus are associated with gene mutation [9,10,11,12]. Therefore, the transformation of non-B structures into canonical B-form DNA is crucial for maintaining genomic integrity and stability, especially in some important regions such as replication origin and transcription start site [13,14]. To achieve this, non-B structures have to be dissociated, and an additional driving force is needed. It has been demonstrated in our previous studies that positive supercoils associated with nucleosome assembly [15] and DNA replication [16] could disintegrate some non-B DNA structures. However, only instantaneous and relatively low-level positive supercoils were generated in those courses. Since reverse gyrase can induce inherent and high-level positive supercoils into DNA substrates [17,18], the disintegrating ability to the non-B DNA structures might be improved.

Among many non-B structures, G-quadruplex is a thermodynamically stable structural entity, and the motifs for the formation of G-quadruplexes widely exists in genomes of various organisms [19,20,21]. More and more studies have shown that G-quadruplex is one of the most important secondary structures playing crucial roles in many significant biological processes such as gene regulation [22,23,24]. G-rich sequences wildly exist in the duplex regions of genomic DNA, where the formation of G-quadruplex was blocked by its complementary strands and also the adjacent duplex regions. However, once it is formed, G-quadruplex structure is very difficult to be disintegrated to restore duplex conformation because of the high thermo-stability of the non-B structure [25]. It has therefore attracted much attention in studying the competition mechanism between duplex and G-quadruplex [26,27,28]. DNA supercoiling, on the other hand, can change the structural property of double helix by unwinding or overwinding DNA double strands [29,30]. In our previous studies, it has been demonstrated that DNA gyrase can facilitate the G-quadruplex generation from plasmid DNA under the intracellular concentrations of potassium ions, and the driving force that makes the G-quadruplex structure formation is presumably the unwinding DNA duplex associated with negative supercoils [31]. In the current studies, on the other hand, the interaction between a pre-formed thermostable G-quadruplex and overwinding DNA double strands caused by reverse gyrase has been investigated. The disintegration of thermal stable G-quadruplex and resumption of the original canonical B conformation were verifiable and analyzable through using atomic force microscopy and its associated software, as well as through electrophoretic analyses and endonuclease digestion assays.

## 2. Results and Discussion

### 2.1. Synthesis and Structural Confirmation of G-Quadruplex-Containing DNA 1

Previous in vitro studies showed that most of the G-quadruplex structures exhibit high melting points and are incapable of resuming their original B-conformation under the physiological conditions once they are formed [32,33]. In addition, the thermodynamic stability of G-quadruplex is closely related with the number of G-quartets [24]. In order to investigate whether positive supercoils affiliated with reverse gyrase can drive the structural transformation of the high thermal stable G-quadruplex into DNA duplex, a G-quadruplex-containing mini-plasmid DNA (DNA 1) was synthesized as the initial DNA substrate for reverse gyrase. The G-quadruplex in DNA 1 was designed to possess six G-quartets, and this structural entity keeps stable attemperatures as high as 90 °C in KCl [33]. (see Table S2)

**Figure 1:**
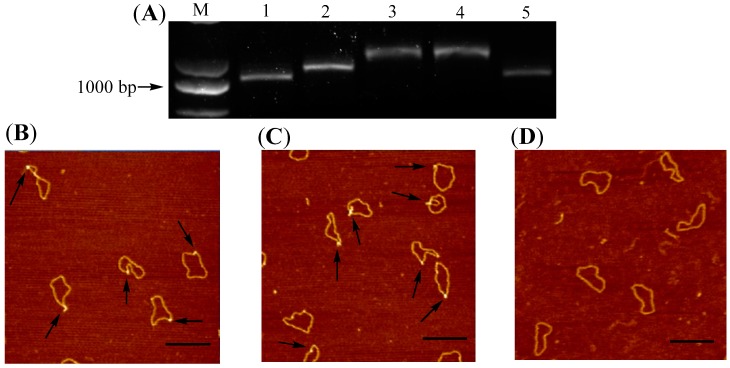
Synthesis and structural examination of DNA 1 and DNA C1. (**A**) Electrophoretic analysis of DNA products involved in synthesizing DNA 1. Lane M: Molecular weight markers; Lane 1: DNA S1; Lane 2: DNA N1 obtained by nicking endonuclease digestion; Lanes 3: DNA 1; Lane 4: DNA samples obtained by heating DNA 1 to 90 °C follow by cooling down to room temperature; Lane 5: DNA C1; (**B**) Structural confirmation of DNA 1 using AFM. The DNA sample used for this AFM examination was the same batch of sample as the one loaded into Lane 3 in Figure 1A; (**C**) Structural confirmation of the integrity of G-quadruplex in DNA 1 after the heat denaturation. The DNA sample used for this AFM examination was the same batch of sample as the one loaded into Lane 4 in Figure 1A; (**D**) Structural confirmation of DNA C1 using AFM. The DNA sample used for this AFM examination was the same batch of sample as the one loaded into Lane 5 in Figure 1A. Scale bar: 200 nm.

The synthetic route toward DNA 1 was given in Figure S1. To construct this G-quadruplex-containing circular DNA, a guanine-rich segment was induced by Polymerase Chain Reactions (PCR) with the particular designed primers (Primer 1 and 2 in Table S1). The circularization of DNA follows our previous reported methods [16] and the mini-plasmid DNA with a guanine-rich segment was obtained (DNA S1, see support information). In order to promote the formation of G-quadruplex in DNA circle, a nick point was created in one strand of DNA duplex (DNA N1), which could prevent from generating torsional constraint when G-quadruplex formed [15]. Compared with other topoisomers, circular DNA with nicked site (s) is a “most” relaxed conformation [34]. However, low level of torsion exists in the strands of covalently closed circular DNA obtained by linear DNA circularization, especially in small circles [35]. As shown in Figure 1A, DNA N1 ran slower than DNA S1, which implied that DNA N1 possesses a more relaxed structure. It is also consistent with our previous report [15]. It has been reported that molecular crowded conditions caused by PEG facilitate the formation of G-quadruplex because of its significant G-quadruplex stabilization and duplex destabilization [26]. DNA N1 was accordingly incubated in PEG 200 and further re-sealed using DNA ligase to give DNA 1. As shown in Figure 1A, the mobility shift of DNA 1 (Lane 3) is slower than the one of DNA S1 (Lane 1). Since the formation of G-quadruplex in DNA circle should in theory alter the entire topological structure of DNA, the observation of slower-moving band in Lane 3 is consistent with the suggestion that non-B DNA structures were produced. With the purpose to test the thermal stability of the newly formed G-quadruplex in DNA circle, the solution containing DNA 1 was heated to 90 °C for 5 min and cooled to room temperature. As shown in Lane 4, there is no mobility shift difference observed in electrophoretic analysis. AFM has been known to be a powerful tool to detect the subtle alteration of biological macromolecules [36,37]. The same batch of DNA samples loaded in Lane 3 and Lane 4 were also examined by AFM. As shown in Figure 1B,C, some raised structures (e.g., spurs and blobs) can be observed in both DNA samples. The observations shown above indicated that the non-B structures in DNA 1 remained stable at 90 °C and cannot transform into duplex, which are also consistent with previous reports [33]. With the aim to confirm that the observed non-B structures are indeed associated with the guanine-rich sequence in DNA 1, DNA C1 was designed and synthesized. The sequence of DNA C1 is identical to DNA 1 except that the G-rich segment was replaced by a different 33 base-paired duplex segment (see Table S3). As shown in Figure 1D, no G-quadruplex structure existed along the duplex backbone of DNA C1.

### 2.2. Introduction of Positive Supercoils and Dissociation of G-Quadruplex Driven by Reverse Gyrase

**Figure 2:**
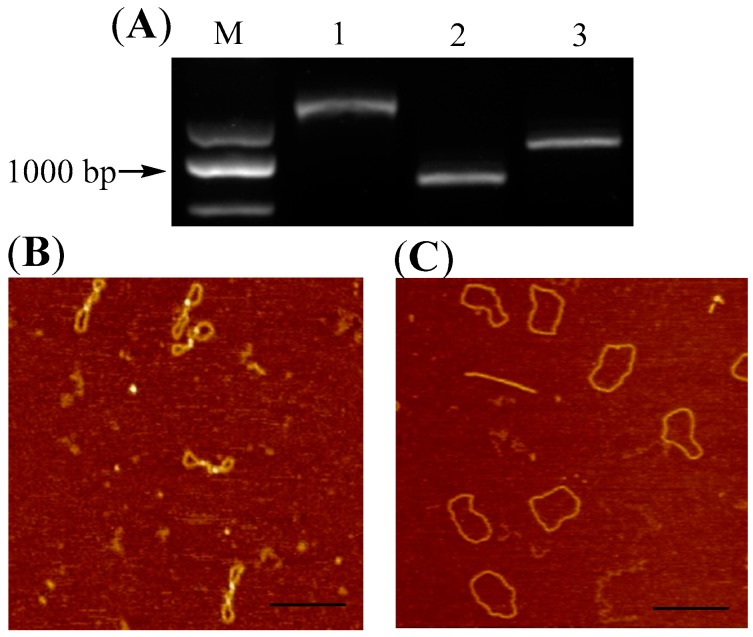
Disintegration of G-quadruplex structures in positively supercoiled DNA molecules. (**A**) Electrophoretic analysis of DNA products after positive supercoils introduction follow by Topo I relaxation. Lane M: Molecular weight markers; Lanes 1: DNA 1 only; Lane 2: positively supercoiled DNA 2 obtained by the treatment of DNA 1 with reverse gyrase; Lanes3: DNA 3 obtained by Topo I relaxation; (**B**,**C**) Structural confirmation of DNA 2 and DNA 3 using AFM. The DNA sample used for those AFM examinations were the same batch of samples as those loaded into Lane 2 and 3 in Figure 2A. Scale bar: 200 nm.

Since reverse gyrase can overwind the DNA double strands, we decided next to treat the newly synthesized G-quadruplex-containing DNA 1 with reverse gyrase. As shown in Figure 2A, a band (DNA 2, Lane 2) that migrated faster than that of DNA 1 (Lane 1) was observed, which signified that the entire topological structure of DNA 1 was changed into a more compact conformation. The same batch of DNA sample loaded into Lane 2 was also examined using AFM. As shown in Figure 2B, molecular architecture of DNA molecules existed in space in a self-twisted fashion, which suggested that positive supercoils were induced by reverse gyrase. It has been well studied that the positively supercoiled DNA molecules are highly interwound. We therefore speculated that the tightening of DNA double helix could provide a driven force, and the thermostable G-quadruplex structure has been disintegrated in DNA molecules shown Figure 2B. In order to visualize and confirm that the G-quadruplex structure in DNA 1 has been transformed into duplex in a more accurate manner using AFM, Topo I was used to remove the supercoils in DNA 2 to produce the relaxed DNA molecules (DNA 3, Lane 3 in Figure 2A) [15,16]. As shown in Figure 2C, no raised structures can be observed along the backbone of circular DNA 3 and all DNA molecues exhibited a homogenousspreads, which implies that no G-quadruplex structurewas left in the final DNA products.

### 2.3. Comparison of Structural Parameters in AFM Images

**Figure 3:**
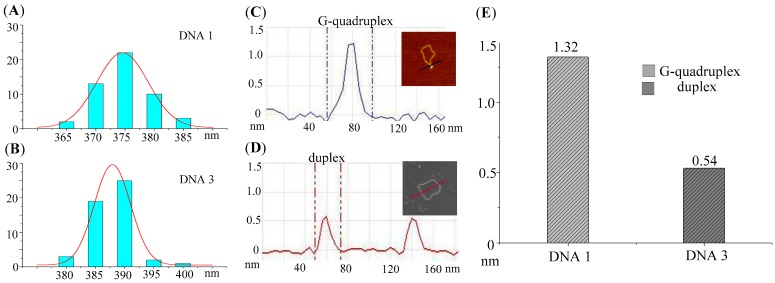
Comparison of the length and height of DNA 1 and DNA 3. (**A**,**B**) Frequency distributions of the lengths (nm) of DNA 1 and DNA 3 in their AFM images. The curves indicate the fitted Gaussian functions; (**C**) Section analyses of a G-quadruplex in DNA 1; (**D**) Section analyses of the two duplex DNA strands in DNA 3; (**E**) Diagrammatic representation the difference between the height of G-quadruplex in DNA 1 and duplex in DNA 3.

It has been reported that disintegration of non-B structures and reformation of duplex can increase the the contour lengths of DNA circle [38,39]. The structural parameters (length and height) of topoisomers (DNA C1, DNA 1, and DNA 3) were measured in order to fruther confirm the G-quadruplexes in DNA 1 has been thanformed into double helix in DNA 3. The lengths (in nm) of DNA were obtained by detecting the circumference alone the backbone of circular DNA, which were measured by drawing a series of very short lines along the DNA contour and summating the lengths [40]. The mean length of DNA C1 (±SE) is 388.23 ± 3.1 nm (*n* = 50) as shown in Table 1. Since there is no G-quadruplex or other apperent non-B forming sequences in DNA C1 (see Table S3 in support information), only duplex conformation can be observated in its AFM images (Figure 1D). The size of DNA C1 is 1132 bp, which will give the nm-to-bp conversion factor to be 0.343 (nm/bp). This factor corresponds to the previous reports of DNA molecules measurement under dry AFM imaging [40]. Mean length of DNA 3 is 387.71 ± 3.0 nm. It is 12.33 nm (equivalent to ~36 bp) longer than the length of G-quadruplex-containing DNA 1 (375.38 ± 3.3 nm), which indicated that 36 base pairs duplex DNA segment in DNA 3 molecules is newly formed and the number is very close to the G-quadruplex forming sequence (33 nt). Frequency distributions of the lengths for DNA 1 and 3 are also shown in Figure 3A,B. These distributions clearly show the increasing of the length of DNA 1 plasmid after reverse gyrase and Topo I treatment.

In addition, the height of G-quadruplex and duplex in DNA 1 and DNA 3 were also measured by section analysis. The raised structures such as spurs and blobs alone the backbone of DNA 1 were frequently observed, which were believed to be the formed G-quadruplex structures. The height measurements were taken across the base of each spur and the middle of each blob. In the case of spurs, all of these structures were included in the dataset because the observed shapes were significantly different from anything seen on pure duplex DNA. On the other hand, small raised structures (blobs) were occasionally seen on pure duplex DNA because of the variations in the imaging surface and/or kinks in the pure duplex DNA. As a result, a criterion was set in order to distinguish the newly formed G-quadruplex structures from the features occasionally found on the pure duplex DNA [40]. The normal height and the peak height were determined for 50 duplex DNA molecules (DNA C1). The mean of normal height was 0.53 ± 0.02 nm, and the mean of peak height was 0.69 ± 0.02 nm, with a highest absolute value of 0.91 nm. Consequently, any blob <1.0 nm in height was excluded from the dataset and any blob ≥1.0 nm was included. As shown in Table 1, about 94 % (n = 50) of the molecules of DNA 1 contained G-quadruplex structures that were not present on the control DNA molecules (DNA C1). After positive supercoils introduction, followed Topo I relaxation, only 4% (n = 50) of the DNA molecules (DNA 3) showed raised structures (all were small blobs). The results signified that almost all the G-quadruplexes in DNA 1 have been transformed into the duplex DNA after treated with reverse gyrase and topo I.

### 2.4. Endonuclease Assays on DNA Topoisomers with or without G-Quadruplex

**Figure 4:**
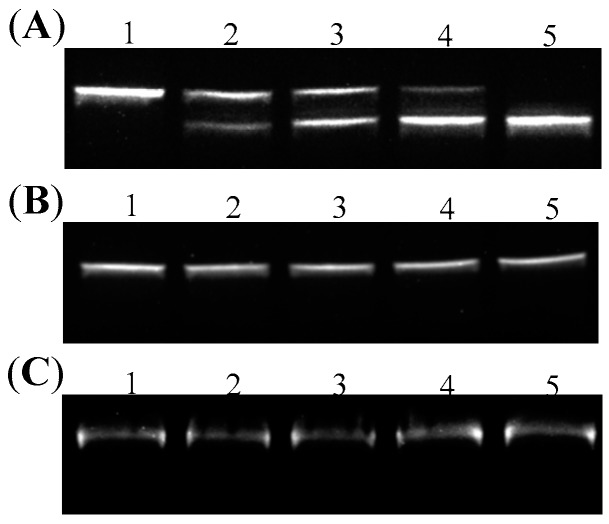
Endonuclease digestion assays to confirm the presence or absence of G-quadruplex structures in DNA topoisomers. Lane 1: Untreated DNA 1 (**A**), DNA 2 (**B**) or DNA 3 (**C**). Lanes 2 to 5: T7 endonuclease I-catalyzed reaction products obtained by incubating DNA 1 (**A**), DNA 2 (**B**) or DNA 3 (**C**) with T7 endonuclease I at 37 °C for 5 min (Lane 2), 10 min (Lane 3), 15 min (Lane 4), and 30 min (Lane 5).

It had been known that the formation of G-quadruplex or other non-B structures can create some non-matched single-stranded regions at the junction of duplex and non-B structures. In order to further confirm the presence of G-quadruplex in DNA 1 and the absence of non-matched regions in DNA 2 and DNA 3 using enzymatic method, T7 endonuclease I, a type of endonuclease that has a special ability to cleave the non-perfectly matched DNA, was used to detect the structural integrity of DNA double helix in the three topoisomers. As shown in Figure 4A, a new band appeared after DNA 1 was treated with T7 endonuclease I, which signified that the non-matched regions existed in DNA 1 and it was cleaved by the endonuclease. On the other hand, no mobility shift difference can be found after 1 h incubation of DNA 2 and DNA 3 with T7 endonuclease I, respectively, as shown in Figure 4B,C. Those observations implied that the duplex conformation in DNA 2 and 3 kept integrate.

### 2.5. A Purposed Mechanism for Disintegration of G-Quadruplex Structures Driven by Reverse Gyrase

**Figure 5:**
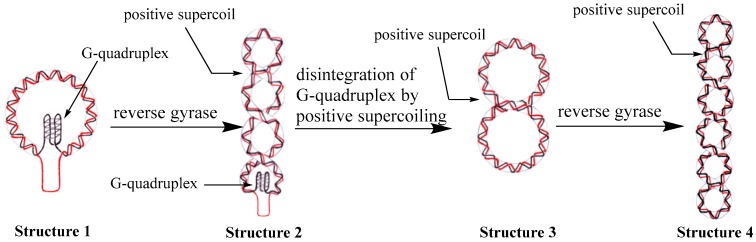
Schematic representation of our proposed pathway of disintegration of G-quadruplex driven by reverse gyrase.

Combining all the evidences shown above, we proposed a possible mechanism for the transformation of thermostable G-quadruplex structures into DNA double helix by reverse gyrase. Figure 5 depicts the anticipated actions of positive supercoiling on a G-quadruplex DNA structure when these two types of structures co-exist in the same duplex DNA strands. Once the G-quadruplex-containing circular DNA (Structure 1) is treated with reverse gyrase, positive supercoils can be induced and the torsional stress will be accumulated in the DNA backbone. At this stage, G-quadruplex structure and DNA supercoils co-exist in the same DNA molecule (Structure 2). Since reverse gyrase can make the double helix of DNA highly interwound, a driven force will be provided to break down the G-quadruplex structure, and the double helical structure could be resumed at the same time. It has been established that restoration of non-matched DNA regions in a covalently closed circular DNA could increase the twist number [34,41]. According to the equation of Lk = Tw + Wr, the positive supercoils will be reduced (Structure 3). Reverse gyrase is a enzyme that can induced certainlevel of positive supercoils into DNA [18]. DNA molecules with Structure 3 and Structure 4 will be recognized by reverse gyrase as the substrates and more positive supercoils can be achieved. The overwinding structure in DNA circles (Structure 4) could prevent the region with G-quadruplex formation sequences to re-generate intra-stranded secondary structures and keep the structural integrity of DNA double helix within the whole DNA molecules.

## 3. Materials and Methods

### 3.1. Reagents and General Information

*Sulfolobus shibatae* was obtained from American Type Culture Collection (51778). Reverse gyrase was purified from *Sulfolobus shibatae* following reported procedures [42]. T7 endonuclease, Nt.BsmAI, T4 DNA ligase, *Taq* DNA polymerase, and SacI were purchased from New England Biolabs (Ipswich, MA, USA). Topo I was provided by Takara Bio Inc. (Shiga, Japan). Primers and plasmid X2420G for PCR were provided by Generay Biotech (Shanghai, China). All the buffer and solution are prepared by the biological purity water.

### 3.2. Reactions of Nicking Endonuclease with Mini-Plasmid DNA

A mixture containing five units of Nt.BsmAI, 1 ×Nt.BsmAI buffer (20 mM Tris-acetate, 50 mM potassium acetate, 10 mM Magnesium Acetate, 1 mM Dithiothreitol), and ~2 μg DNA S1 was incubated at 37 °C for 1 h to generate a nicked site-containing circular DNA (DNA N1). Note: Nt.BsmAI is a nicking endonuclease that cleaves only one strand of duplex DNA, and there is only one binding site for Nt.BsmAI on substrate DNA.

### 3.3. G-Quadruplex Formation from Duplex under Molecular Crowded Condition

A nicked site- and G-quadruplex-containing circular DNA obtained by incubation of DNA N1 in 10 mM Tris-HCl (pH 7.4) buffer containing 1 mM EDTA, 150 mM KCl and 40% PEG 200 at 95 °C for 5 min followed by cooling the mixture to room temperature.

### 3.4. Ligation Reaction for Sealing the Nicking Site

A mixture containing 50 mM Tris-HCl, 10 mM MgCl_2_, 1 mM ATP, 10 mM dithiothreitol, 20 units T4 DNA ligase, and ~500 ng nick-containing DNA was incubated at 16 °C for 8 h to give the DNA products (DNA 1).

### 3.5. Reactions of Reverse Gyrase with Mini-Plasmid DNA

500 ng mini-plasmid DNA was added into a 50 μL solution containing 50 mM Tris-HCl (pH = 8.8), 10 mM MgCl_2_, 1 mM Dithiothreitol, 90 mM NaCl, 30 μg/mL BSA, 1 mM ATP, 1 mM spermidine, and 5 μL solution containing purified reverse gyrase. The mixture was incubated at 75 °C for 0.5 h. After that, 1% SDS, 0.2 mM EDTA, and 1 mg/mL proteinase K were added, and the reaction mixture was incubated at 50 °C for another 0.5 h.

### 3.6. Reactions of Topo I with Supercoiledmini-Plasmid

A 50 μL solution containing 35 mM Tris-HCl (pH = 8), 72 mM KCl, 5 mM MgCl_2_, 5 mM DTT, 5 mM spermidine,0.1% bovine serum albumin (BSA), 500 ng mini-plasmid, and 0.5 U Topo I was incubated at 37 °C for 0.5 h. 

### 3.7. Reactions of T7 Endonuclease I with Supercoiled-Plasmid

A 50 μL solution containing 50 mM NaCl, 10 mM Tris-HCl (pH = 7.9), 10 mM MgCl_2_, 1 mM DTT, 500 ng mini-plasmid DNA, and 0.5 U T7 endonuclease I was incubated at 37 °C for 5 to 30 min.

### 3.8. Gel Electrophoresis

The mobility shift difference of DNA molecules were determined by one-dimensional electrophoresis through 1.5% (*wt*/*vol*) agarose gels run at 3 V/cm in 90 mM Trisborate/2.5 mM EDTA, pH 8.3, for 3 h at room temperature. The direction of electrophoresis was from above to bottom. The gels were incubated in 50 ng of ethidium bromide per mL for 1.5 h after electrophoresis. The gel was photographed using Gel Documentation System (BioRad ChemiDocXRS, Hercules, CA, USA).

### 3.9. Experimental Procedures for DNA Sample Preparations and AFM Examination

AFM examination was conducted following reported procedures and a specially prepared mica surface was selected as the substrates for DNA binding. Generally, the micas used in the our studies were modified on their surfaces with (3-aminopropyl) triethoxysilane (APS) [43]. Sample preparation procedures are described as follows: 5 μL to 10 μL of solutions containing 20 mM Tris-HCl (pH = 7), and 0.1 to 0.01 μg/mL DNA were dropped into the middles of the newly prepared APS-mica plates (1 × 1 cm^2^), which were further kept at room temperature for 5 min. Then, 10 mL of distilled water were then used to rinse the APS-mica plates that has been bound by DNA molecules for three times in order to remove the salt and buffer. Before scanning, the samples were placed in vacuum desiccator for 30 min. AFM images of DNA molecules on the APS-mica plates were obtained in Tapping Mode™ on a Dimension Edge™ AFM (Bruker, Santa Barbara, CA, USA) in connection with a Nanoscope VIII™ controller. Aluminum reflective coating cantilevers with nominal spring constants between 1 and 5 N/m were selected. Scan frequency was 1.9 Hz per line and the modulation amplitude was in a nanometer range. All DNA sample determinations were carried out in air at room temperature. 

## 4. Conclusions

In conclusion, a circular DNA–containing thermal stable G-quadruplex structure was synthesized. It is shown that reverse gyrase can induce a high level of positive supercoils, which could be utilized to make the thermal stable G-quadruplex transformed into its duplex conformation. The DNA molecules with or without G-quadruplex in different topological status were examined by electrophoretic analyses and endonuclease digestion assays, as well as using atomic force microscopy and its associated software. The new observations could imply that, as a hyperthermophile-exclusive topological enzyme, reverse gyrase may be utilized to remove some pre-formed thermal stable non-B structures and protect their DNA from the hot environments in which they live.

## Figures and Tables

**Table 1:** Quantitation of length and height on DNA circles.

Mini-Plasmid	Plasmids with G-Quadruplex (%)	Contour Lengtha	Height of Duplex	Height of G-Quadruplex	N
DNA 1	94	375.38 ± 3.3 nm	0.54 ± 0.02 nm	1.32 ± 0.04 nm	50
DNA 3	4	387.71 ± 3.0 nm	0.54 ± 0.02 nm	N.A.	50
DNA C1	0	388.23 ± 2.1 nm	0.53 ± 0.02 nm	N.A.	50

## Supplementary Materials

The following are available online, Figure S1: Schematic illustration of our synthetic route toward DNA 1, Table S1: Nucleotide sequences of primers used in our polymerase chain reactions, Table S2: Nucleotide sequences of DNA 1, Table S3: Nucleotide sequences of DNA C1.
