# Supplementary material for: Transformation of a Thermostable G-Quadruplex Structure into DNA Duplex Driven by Reverse Gyrase
Source: Molecules. 2017 Nov 22;22(11):2021. doi: 10.3390/molecules22112021 (PMC6150213; doi:10.3390/molecules22112021)
Supplement: Supplementary file 1 [file molecules-22-02021-s001.pdf]

Supporting Information

Transformation of a thermostable G-quadruplex structure into DNA double helix driven by reverse gyrase

Dawei Li[a,*], Qiang wang[a], Yun Liu[a], Kun Liu[b], Qiang Zhuge[a] and Bei Lv[b,*]

[a] *Key Lab of Forest Genetics and Biotechnology, Nanjing Forestry University, 159 Longpan Road, Nanjing, 210037, China*

[b] *Jiangsu Key Laboratory for Biofunctional Molecules, College of Life Science and Chemistry, Jiangsu Second Normal University, Nanjing, 210037, China*

* Corresponding author. Tel.: +86-25-8542-8701; fax: +86-25-8542-8701; e-mail: dwli@njfu.edu.cn or lvbei@jssnu.edu.cn

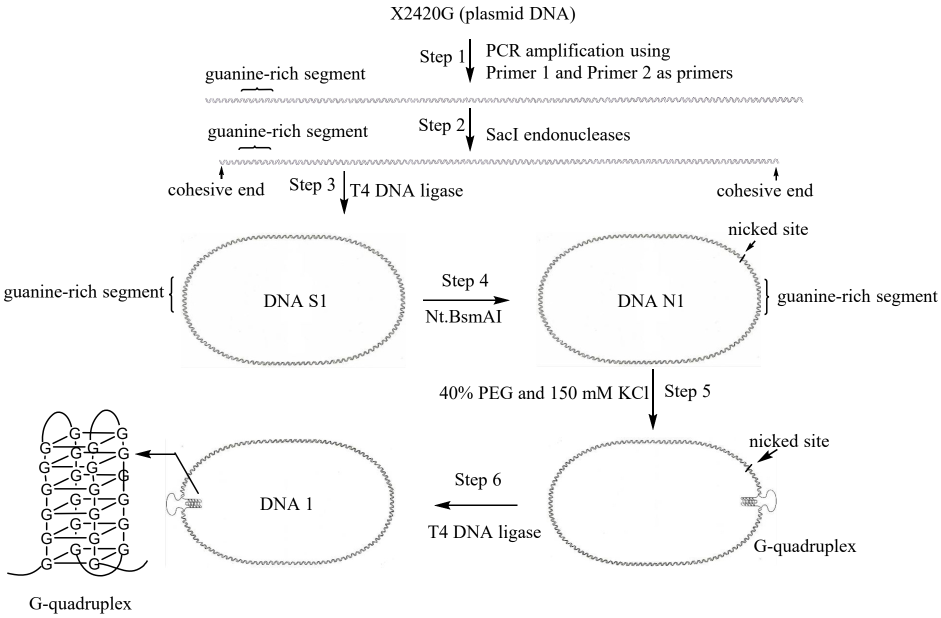

Figure S1 Schematic illustration of our synthetic route towards DNA 1.

Experimental procedures:

Step 1: X2420G (plasmid DNA) was purchased from Generay Biotech (Shanghai, China). The forward primer (Primer 1 in Table S1) contained the cytosine-rich segment. The detailed nucleotide sequences of Forward Primer (Primer 1) and reverse primer (Primer 2) used in the current studies are shown in Table S1. The PCR amplification reactions were carried out following reported procedures with a annealing temperature of 63 °C.

Step 2: A mixture containing 10 mM Bis-Tris-Propane-HCl, 10 mM $MgCl_2$, 1 mM Dithiothreitol, 10 units SacI and ~2 μg purified PCR products was incubated at 37 °C for 1 hour, which gave rise to a cohesive end-containing linear DNA.

Step 3: A mixture containing 50 mM Tris-HCl, 10 mM $MgCl_2$, 1 mM ATP, 10 mM dithiothreitol, 20 units T4 DNA ligase and ~2 μg SacI digested products was incubated at 16 °C for 8 hours. The

resultant reaction mixture was allowed next to react with BAL-31 (an exonuclease that hydrolyzes opening end-containing DNA) in order to acquire pure closed circular DNA products (DNA S1).

Step 4: A mixture containing 5 units of Nt.BsmAI, 1 x Nt.BsmAI buffer (20 mM Tris-acetate, 50 mM potassium acetate, 10 mM Magnesium Acetate, 1 mM Dithiothreitol) and ~2 μg DNA S1 was incubated at 37 °C for 1 hour to generate a nicked site-containing circular DNA (DNA N1).

Step 5: A nicked site- and G-quadruplex-containing circular DNA obtained by incubation of DNA N1 in 10mM Tris-HCl (pH 7.4) buffer containing 1mM EDTA, 150 mM KCl and 40% PEG 200 at 95 °C for 5 minutes followed by cooling the mixture to room temperature.

Step 6: A mixture containing 50 mM Tris-HCl, 10 mM $MgCl_2$, 1 mM ATP, 10 mM dithiothreitol, 20 units T4 DNA ligase and ~500 ng DNA products obtained in Step 5 was incubated at 16 °C for 8 hours to give the final DNA products (DNA 1)

Table S1. Nucleotide sequences of primers used in our polymerase chain reactions.

Name of DNA	Nucleotide sequence
Primer 1	5′CCGAGCTCAGGACCCCCCATTCCCCCCATTCCCCCCATTC CCCCCTAATACATGTGCTGAGGATCGAG 3′
Primer 2	5′ TCGTTTGGTATGGCTTCATT 3′
Primer 3	5′CCGAGCTCAGGACATAACATTCTGCCCATTCCTTTCATTC CGGTCTAATACATGTGCTGAGGATCGAG 3′

Table S2 Nucleotide sequences of DNA 1. Sequence only shows one strand from 5' to 3'. Gray shadow indicates the sequence that can form the G-quadruplex structures.

```
<5'GAGCTCCAGCTCTTCTCTCCTTCGATCAAGGCGAGTTACATGATCCCCCATGTTGTGCAAAAAGCTTAACTT
ACCGCTGTTGAGATCCAGTTCGATGTAACCCACTCGTGCACCCAACTGATCTTCAGCATCTTTTACTTTCACCAGC
GTTTCTGGGTGAGCAAAAACAGGAAGGCAAAATGCCGCAAAAAAGGGAATAAGGGCGACACGGAAATGTTGAATAC
TCATACTCTTCCTTTTTCAATATTATTGAAGCATTTATCAGGGTTATTGTCTCATGAGCGGATACATATTTGAATG
TATTTAGAAAAATAAACAAATAGGGGTTCCGCGCACATTTCCCCGAAAAGTGCCACCTGACGTCTAAGAAACCATT
ATTATCATGACATTAACCTATAACAAGAATTCTCATGTTTGACAGCTTATATAACTTCGTATAATGTATGCTATAC
GAAGTTATGGCTCGAGACCGGTTCTAGATACCTAGGTTGGTACCCTCTAGTCAAGGCCTTAAGTGAGTCGTATTAC
GGACTGGCCGTCGTTTTACAACGTCGTGACTGGGAAAACCCTGGCGTTACCCAACTTAATCGGTTTTGCGACGAGG
ATCCACTAAGTGGGAAGACTTGCCGAATTCCTTCAAGCCTGCTTTTTTGTACAAAGTTGGCATTATAAAAAAGCAT
TGCTCATCAATTTGTTGCAACGAACAGGTCACTATCAGTCAAAATAAAATCATTATTTGGGGCCCGAGCTTAAGAC
TGGCCGTCGTTTTACAACGTCGTGACTGGGAAAACATCCATGCTAGCGTTAACGCGAGAGTAGGGAACTGCCAGGC
ATCAAATAAAACGAAAGGCTCAGTCGGTAGACTGGGCCTTTCGTTTTATCTGTTGTTTGTCGGTGAACGCTCTCCT
GAGTAGGACAAATCCGCCGGGAGCGGATTTGAACGTTGTGAAGCAACGGCCCGGAGGGTGGCGGGCAGGACGCCCG
CCATAAACTGCCAGGCATCAAACTAAGCAGAAGGCCATCCTGACGGATGGCCTTTTTGCGTTTCTACAAGGCCGGC
AGTAATTAAGACTCGATCCTCAGCACATGTATTTAGGGGGGAATGGGGGGAATGGGGGGAATGGGGGGTCCT
3'>
```

Note: (1) DNA 1 is a circular DNA; and

(2)< and > stand for the termini that are covalently connected.

Table S3 Nucleotide sequences of DNA C1. Sequence only shows one strand from 5' to 3'. Gray shadow indicates the sequence that are different from DNA 1 and cannot form G-quadruplex.

```
<5'GAGCTCCAGCTCTTCTCTCCTTCGATCAAGGCGAGTTACATGATCCCCCATGTTGTGCAAAAAGCTTAACTT
ACCGCTGTTGAGATCCAGTTCGATGTAACCCACTCGTGCACCCAACTGATCTTCAGCATCTTTTACTTTCACCAGC
GTTTCTGGGTGAGCAAAAACAGGAAGGCAAAATGCCGCAAAAAAGGGAATAAGGGCGACACGGAAATGTTGAATAC
TCATACTCTTCCTTTTTCAATATTATTGAAGCATTTATCAGGGTTATTGTCTCATGAGCGGATACATATTTGAATG
TATTTAGAAAAATAAACAAATAGGGGTTCCGCGCACATTTCCCCGAAAAGTGCCACCTGACGTCTAAGAAACCATT
ATTATCATGACATTAACCTATAACAAGAATTCTCATGTTTGACAGCTTATATAACTTCGTATAATGTATGCTATAC
GAAGTTATGGCTCGAGACCGGTTCTAGATACCTAGGTTGGTACCCTCTAGTCAAGGCCTTAAGTGAGTCGTATTAC
GGACTGGCCGTCGTTTTACAACGTCGTGACTGGGAAAACCCTGGCGTTACCCAACTTAATCGGTTTTGCGACGAGG
ATCCACTAAGTGGGAAGACTTGCCGAATTCCTTCAAGCCTGCTTTTTTGTACAAAGTTGGCATTATAAAAAAGCAT
TGCTCATCAATTTGTTGCAACGAACAGGTCACTATCAGTCAAAATAAAATCATTATTTGGGGCCCGAGCTTAAGAC
TGGCCGTCGTTTTACAACGTCGTGACTGGGAAAACATCCATGCTAGCGTTAACGCGAGAGTAGGGAACTGCCAGGC
ATCAAATAAAACGAAAGGCTCAGTCGGTAGACTGGGCCTTTCGTTTTATCTGTTGTTTGTCGGTGAACGCTCTCCT
GAGTAGGACAAATCCGCCGGGAGCGGATTTGAACGTTGTGAAGCAACGGCCCGGAGGGTGGCGGGCAGGACGCCCG
CCATAAACTGCCAGGCATCAAACTAAGCAGAAGGCCATCCTGACGGATGGCCTTTTTGCGTTTCTACAAGGCCGGC
AGTAATTAAGACTCGATCCTCAGCACATGTATTTAGACCGGAATGAAAGGAATGGGCAGAATGTTATGTCCT
3'>
```

Note: (1) DNA C1 is a circular DNA; and

(2)< and > stand for the termini that are covalently connected.
